# Genepi: a blackboard framework for genome annotation

**DOI:** 10.1186/1471-2105-7-450

**Published:** 2006-10-12

**Authors:** Stéphane Descorps-Declère, Danielle Ziébelin, François Rechenmann, Alain Viari

**Affiliations:** 1 GENOME express, Meylan, France; 2 Université Joseph Fourier, Grenoble, France; 3 INRIA Rhône-Alpes, Helix group, Montbonnot, France

## Abstract

**Background:**

Genome annotation can be viewed as an incremental, cooperative, data-driven, knowledge-based process that involves multiple methods to predict gene locations and structures. This process might have to be executed more than once and might be subjected to several revisions as the biological (new data) or methodological (new methods) knowledge evolves. In this context, although a lot of annotation platforms already exist, there is still a strong need for computer systems which take in charge, not only the primary annotation, but also the update and advance of the associated knowledge. In this paper, we propose to adopt a blackboard architecture for designing such a system

**Results:**

We have implemented a blackboard framework (called Genepi) for developing automatic annotation systems. The system is not bound to any specific annotation strategy. Instead, the user will specify a blackboard structure in a configuration file and the system will instantiate and run this particular annotation strategy. The characteristics of this framework are presented and discussed. Specific adaptations to the classical blackboard architecture have been required, such as the description of the activation patterns of the knowledge sources by using an extended set of Allen's temporal relations. Although the system is robust enough to be used on real-size applications, it is of primary use to bioinformatics researchers who want to experiment with blackboard architectures.

**Conclusion:**

In the context of genome annotation, blackboards have several interesting features related to the way methodological and biological knowledge can be updated. They can readily handle the cooperative (several methods are implied) and opportunistic (the flow of execution depends on the state of our knowledge) aspects of the annotation process.

## Background

The first complete genomic sequence of a living organism, the bacterium *H. influenzae*, was obtained in 1995. Ten years after, the number of fully sequenced genomes is steadily increasing: more than 350 bacterial and archeabacterial genomes and 20 eukaryotic genomes are presently available in public databases. However, the availability of the sequence is merely a starting point. The real challenge actually consists in interpreting and annotating the genomic text. When annotating a genome, biologists are especially looking for the genes, i.e. the regions of the chromosome containing the information to produce proteins or RNA, as well as regulatory signals. Finding all genes and regulatory signals on a complete raw genomic sequence is still an open problem, especially in the case of eukaryotic genomes where the coding regions are interspersed with non-coding regions called introns. Moreover, finding genes and signals is just the first step of the process. Once this has been done, the biologist should face the question to assign a putative function to the gene's product. This is done, for instance, by scanning databases of known proteins in order to pickup those that most resemble the protein to identify. Finally, once all these information have been collected, new and more complex questions arise, such as positioning the protein within its metabolic or gene regulation networks. All these steps compose the annotation process and involve computer programs as well as a lot of human expertise.

Genome annotation can be seen as an incremental, cooperative, data-driven, knowledge-based process [1]. The prediction of coding regions and regulatory signals requires the application of multiple methodsand the adequate combination of their results [2]. For instance, Markov chain models can be used to compute the protein-coding probability of a genomic region [3] and pattern matching or statistical methods will help locating intron-exon junctions and signals. Moreover, results of proteic or nucleic database scanning will usually be superimposed to these predictions, together with any kind of additional information supporting the predicted gene structure. Biologists will retain a gene prediction by confronting these various results and adding their own expertise.

The annotation is thus a long and tedious interpretation process. Moreover, it might have to be executed more than once and subjected to revisions. First of all, sequencing errors may be reported or manual corrections may be supplied by experts and will ask for the reannotation of the corrected regions. Moreover, as new prediction methods appear, they should be applied on the already annotated genomes to produce up-to-date annotations. There is therefore a strong need for computer systems which take in charge, not only the primary detection of genomic features, but the whole incremental annotation process. In this paper, we propose to adopt a blackboard architecture for designing such a system.

### Related works

To our knowledge, no annotation software has ever been designed as a blackboard system, but several existing automatic annotation platforms have adopted well-recognized architectures. Our purpose is not to list hereafter all the existing platforms (for reviews see [4,5]), but to pinpoint those that are emblematic of a particular problem-solving architecture.

Biopipe [6] has been partly inspired from Ensembl [7]. It organizes the annotation methods into pipelines. In Biopipe, a pipeline is the association of:

• A set of "analyses", which describe how a method can be accessed and what are the adequate parameter values;

• A set of rules, which specify when and how a method has to be executed; the set of rules thus defines the possible sequences of analysis methods;

• A manager, which is in charge of accessing the data.

Taverna [8] has adopted a workflow architecture, which adds control structures to the pipeline architecture. It is thus possible to specify iteration loops or conditional alternatives over the methods. Taverna offers graphical interfaces to describe the graph which links the "processors", i.e., the data transformation methods. External methods can be remotely called as Web services.

In the ImaGene [9] object-oriented system, the sequence analysis methods are organized into tasks. A task is described by the objects it accepts as input and the objects it creates as output. Tasks can be arranged into more complex tasks with the help of operators: sequence (thus allowing the description of pipe-lines), conditional alternative, iteration. The user can follow the successive steps of execution of a complex task: its actual flow of execution is displayed together with its structure. Executions are themselves objects and can be stored and executed again, possibly after modifying some parameter values.

In rule-based systems, the biological data are represented by facts and the methodological knowledge is represented by rules. Rules express how facts in the current state of the system allow to infer new facts, which in turn allow the activation of rules, and so on. MagPie [10] is a good example of such a rule-based annotation system. Two sets of Prolog rules run concurrently. The first one is dedicated to the data collection task; the second to the analysis task. The rules dictate the sequence of execution of the elementary methods, which consume and produce data as Prolog assertions.

In GeneWeaver [11], the annotation system results from the interactions between autonomous, but interacting agents. Five classes of agents are identified:

• The Primary database agents maintain a shared sequence database up to date so that it can be read and used by the agents which need these information;

• The Non-redundant database agents rely on the information provided by the Primary database agents to maintain a curated non-redundant database.

• The Genome agents manage the information related to a particular genome;

• The Calculation agents are associated to sequence analysis methods;

• The Broker agents register and manage the information on all the other agents so as to facilitate their working.

One of the limitations of GeneWeaver is that it has never actually been deployed as a truly operational annotation system. However it recently gave rise to AGMIAL [12], a system actually used in microbiology laboratories.

Other systems using multi-agent concepts have been proposed: including BioMAS/DECAF [13] and EDIT_ToTrEMBL [14]. The BioMAS agents, for instance, are classified into three main categories:

• The Information Extraction agents provide access to databases as well as some calculation services (such as sequence similarity search or feature predictions)

• The Task agents are mostly generic middle agents except for the Annotation Agent that orchestrates the collection of information for each sequence and, therefore, provides some reasoning capabilities about sequence features.

• The interface agents communicate with other agents and provide user interface to manual annotation and database querying.

These various architectures can be classified into two main categories. The first category, which includes pipeline, workflow and task-based systems, is characterized by a sequential method invocation scheme. The second class, which includes multi-agent, blackboard-based and rule-based systems, is characterized by an opportunistic, event-driven, method invocation scheme. The advantages and drawbacks of these various approaches will be discussed later on in the Discussion section.

### The blackboard architecture

Blackboard systems exhibit several similarities with aforementioned rule-based systems: a shared working memory, a procedural representation of knowledge and an inference cycle. The blackboard architecture is well known to support cooperative data-driven interpretation processes [15]. Indeed, the first experiment in blackboard development resulted in the Hearsay system dedicated to human speech understanding [16]. The input to the system is the signal of a microphone; the output is expected to be a database query as it was expressed by the speaker.

**Figure 1:**
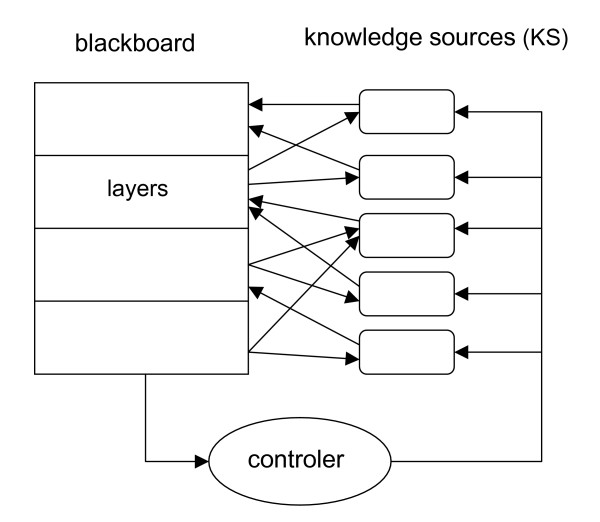
**The blackboard architecture**. The blackboard itself is a shared working space organized into layers. At any time during the problem solving process, it contains all the objects that have been either entered as data or inferred through the execution of knowledge sources. Knowledge sources take as input objects on one or several specific layers and write down the inferred objects on other upper layers. Among all the knowledge sources that could be applied at a given time, the controller selects the ones that will be actually executed.

A blackboard system has three main components [17]: the blackboard itself, a set of knowledge sources, and the controller (Figure 1).

#### The blackboard and the layers

The blackboard is a shared working space, hierarchically structured into layers. Each layer receives domain entities. These entities are produced by knowledge sources acting on entities belonging to lower layers. The bottom layer is directly populated with input data. In Hearsay, the bottom layer contains the raw signal coming from the microphone; the second layer receives the segments into which this continuous signal was decomposed; the third layer stores the hypothetical phonemes associated to each segment; on the fourth layer, these phonemes are grouped into syllables and so on, up to the last layer which contains the formal database query.

Since a knowledge source may produce more than one entity for a given set of input data, the entities are given an hypothesis status. Some of them will be confirmed later on and merged into new entities stored in upper layers. Some others will be further discarded. The management of hypotheses is therefore an important feature of a blackboard system.

#### The knowledge sources

All layers of the blackboard are observed by knowledge sources (KS). A KS takes as input the entities of one or more layers and will infer new entities to be stored on one or more higher layers. Inference of a new entity may be the result of an algorithm, a set of expert rules, a formal neural network, or any executable code. From the system point of view, a KS is actually a black box which is only known by the pattern of entities it expects as input (the activation condition) and the type of entities its produces as output. In Hearsay, the KS working on the lowest layer is a signal processing method; other KSs deal with the succession of phonemes and attempt to merge them into syllables; other KSs look up lexicons to predict words from syllable or check the syntax of a predicted sentence.

#### The controller and the inference cycle

The inference process of a blackboard system follows a cycle. First, as an event, such as the creation of an entity, occurs on the blackboard, the controller will inform all the KSs that are concerned by this event. Each selected KS then checks if its activation condition is satisfied or not. The resulting list of activable KSs is further sorted by the controller in order to prioritize the KSs that must be activated first. The further execution of these KSs will then produce new entities on the blackboard, thus triggering new events and a new cycle begins. The process ends when no KS can be further activated so that the state of the blackboard remains unchanged. The role of the controller is essential in focusing the inference process. It maintains an agenda of the pending KSs and may change the priorities of the agenda entries according to any specified criteria. The efficiency of the overall problem solving process may strongly depends upon the strategy used by the controller to order the KSs.

#### Adequacy of the blackboard architecture to problem solving

Born as an AI architecture, the blackboard presents indeed several interesting features from the knowledge and software engineering points of view. Most of these properties derive from the existence of a shared working space, which represents, at any time, the state of the system. First of all, the KSs do not interact with each other. A KS is only concerned by the events occurring on one (or more) layer(s). At this time, it checks whether some patterns of entities match its own input pattern and declares itself as applicable to the controller. It is eventually executed when requested by the controller and finally writes its results onto its associated output layers. A KS can therefore be added or removed from the overall system without affecting the other KSs. Moreover, the inner part of a KS can be modified without affecting the system, as long as its activation pattern is not modified. Alternatively, the strategy of the controller can be independently modified and tuned up in order to make the inference process more efficient.

The inference process is said to be opportunistic: the sequence of method invocations is not explicitly expressed before run time, but is decided according to the state of the system. As a striking illustration of this opportunistic behavior, if data produced by external sources are laid onto the blackboard, they are taken into account as if internally produced by the KSs and will affect the inference process accordingly.

Blackboard systems have been built for a large spectrum of applications, for which the problems to be solved could not be linearly or hierarchically decomposed into sub- problems. This is the case of most interpretation problems, such as the seminal example of speech analysis and understanding. Examples of application of blackboards in biology include Protean [18], which was designed to predict the 3D conformation of a sequence of amino acids through the application of known physical and chemical constraints, and Crysalis [19], which was designed to analyze the data resulting from the X-ray diffraction pattern of a protein in order to reconstruct its 3D structure.

#### Adequacy of the blackboard architecture for genome annotation

Apart from the striking similarities between genomic sequence annotation and speech analysis, the decision to adopt a blackboard architecture for an annotation system has been motivated by the very nature of the annotation process. As explained previously, the annotation process relies on multiple methods, the execution of which provides different clues on the presence of coding sequences and regulatory signals. These clues have to be confronted, possibly discarded, but hopefully merged at different levels to finally predict the location and the structure of genes. The annotation process can thus be seen as a cooperative (several methods are implied), opportunistic (the flow of execution depends on the state of the blackboard, i.e. on the results of the previously executed KS), knowledge-based (the conditions under which a method may be applied has to be explicitly expressed) and data-driven (the problem solving process is directed by the occurrence of patterns on the input DNA sequence) problem solving process.

This latest point is probably the most important and the most characteristic of blackboard systems as compared to more traditional architectures. In blackboard systems each KS is autonomous and responsible of recognizing a particular state of the shared working space (its activation pattern) and declaring itself as activable. Therefore, the sequence of execution of methods is not programmed in a procedural manner but depends upon the current state of the blackboard. This aspect sometimes causes trouble to developers who want to have full control on the sequence of execution. With blackboards, one should better think in terms of event-driven programming.

## Implementation

**Figure 2:**
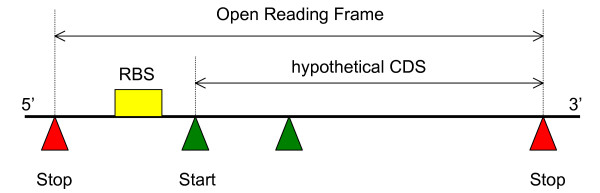
**A basic strategy to look for prokaryotic codingsequence (CDS)**. A coding sequence is known to start with one Start codon (ATG, GTG or TTG) and ends with a Stop codon (TAA,TAG,TGA) in the same frame (they are separated by a multiple of 3 bases). In-frame Start codons may also appear within the CDS, in which case they code for the methionine. A basic searching strategy therefore consists in first identifying ORFs (Open Reading Frames), i.e. regions which are delimited by two in-frame Stop triplets and long enough to code a protein (typically containing more than 150 bases). The heuristics then searches for the leftmost in-frame Start triplet (i.e. the one yielding the longest predicted CDS). The further discovery of a pattern associated to a ribosome binding site (RBS) upstream of the CDS (usually less than 10 nucleotides before Start) will ascertain this CDS. Conversely, the presence of an RBS inside (but not too far from the beginning) a CDS may lead to the selection of an other Start. Finally, the retrieval of similar sequences in annotated sequence databases may eventually lead the biologist to assert the presence of a coding region. This strategy is given only for illustrative purpose.

We have implemented a blackboard framework (called Genepi) for developing automatic annotation systems. By framework, we mean that the system is not bound to any specific annotation strategy or to any particular KSs. Instead, the user can specify a blackboard structure (layers and KSs) in a single configuration file and the system will actually instantiate and run this particular annotation strategy. This allows designing several strategies to target specific biological applications. However, all these strategies will share common mechanisms and properties that will be described by using the very simple prokaryotic annotation strategy depicted in Figure 2. The strategy consists in first identifying Open Reading Frames (ORFs), i.e. regions which are delimited by two in-frame Stops, and then to search for the leftmost in-frame Start triplet within each ORF. The observation of a ribosome binding site pattern (RBS) upstream of the Start triplet will ascertain the CDS [20]. Finally, the retrieval of similar sequences in annotated sequence databases may eventually lead the biologist to annotate the CDS. This strategy is given mainly for the purpose of illustration; a more sophisticated and realistic example will be presented later in the Results section.

### The blackboard, the layers and the KSs

**Figure 3:**
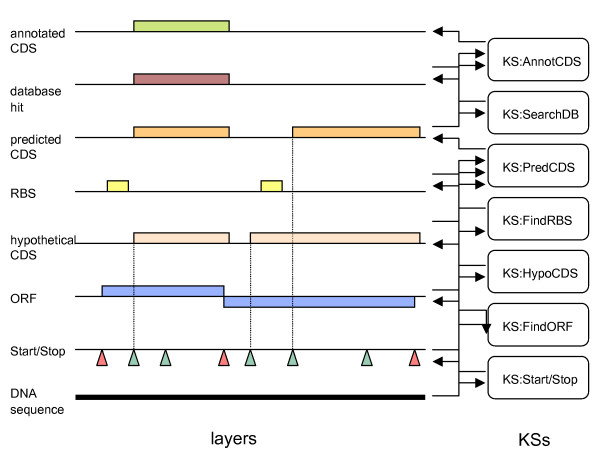
**Example of layers and knowledge sources implementing the basic annotation strategy of Figure 2**. The layers are named after the type of objects they accommodate. Knowledge sources take objects from one or several layers as input and produce new objects on upper layers. *KS:Start/Stop *and *KS:FindRBS *directly work on the raw DNA sequence to locate respectively the Start and Stop triplets and the ribosome binding sites (RBS). *KS:FindORF *builds ORFs from the Starts and Stops locations. KS:HypoCDS computes a *hypothetical CDS *by retaining the leftmost in-frame Start within an ORF. KS:predCDS validates a hypothetical CDS (or possibly modifies its beginning) because of the existence of an RBS. The resulting CDS is placed on the *predicted CDS *layer. The final step consists in searching sequence databases for similarities with known genes to finally ascertain *annotated CDS*s.

The blackboard itself is structured into a hierarchy of layers, all collinear with the input sequence (Figure 3). The lowest layer contains the genomic sequence to be analyzed. The highest one contains the annotated genes as predicted by the application of the various knowledge sources. Each layer contains genomic features (hereafter simply called "features") that have either been detected on the raw sequence or built through the aggregation of features from lower layers. A genomic feature, such as a *Stop triplet*, a ribosome binding site (*RBS*) or a coding region (*CDS*), is described by its origin, its end and its type, *i.e*. as an oriented and typed interval over the sequence.

### The KS activation patterns

**Figure 4:**
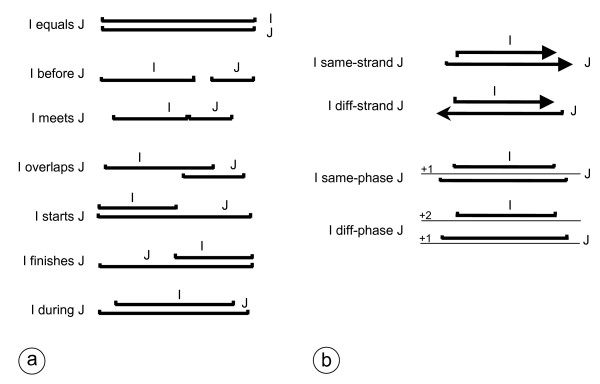
**Allen's set of relations and their extension for the expression of KS patterns**. I and J are two features (intervals) defined by their type and the position of their beginning and end on the sequence. (a) Allen's original relations. (b) extensions of the Allen's set of relations to take into account two specificities of intervals over DNA sequences. **same-strand (I, J) **holds if I and J are on the same strand; **diff-strand (I, J) **holds if I and J are on opposite strands. **same-phase (I, J) **holds if I and J are in the same phase (i.e. they are on the same strand and they are separated by a multiple of three bases); **diff-phase (I, J) **holds if I and J are on different phases.

Some KSs integrate feature detection methods relying on bioinformatics algorithms such as Markov modeling or pattern searching, while others merely confront and merge features into more complex structures. An example of the latter is *KS:HypoCDS *in Figure 3. This KS computes an *hypothetical CDS *starting from an *ORF *and an in-frame *Start *triplet located within this *ORF*. More generally, it turns out that a large number of such annotation rules can be expressed by considering the relative position of the intervals representing the features. That is, by considering, for instance, that a given interval is located "before" or "after", or "overlaps" another interval. More formally, the activation pattern of a KS can be expressed by using a set of relations between intervals adapted from Allen's work on temporal relations [21] (Figure 4a), completed with four relations specific to genomic sequences (Figure 4b). Figure 3 provides several examples of these extended Allen relations in activation patterns. For instance, the activation pattern of *KS:HypoCDS *states that this source should be activated when it exists an in-frame *Start *"during" an *ORF*. In the same way, *KS:AnnotCDS *will be activated when it exists a *database hit *"during" a predicted CDS. This KS will further ensure that the characteristic of the overlap (e.g. the sequence identity) is sufficient to produce an *annotated CDS *in the upper layer. When the activation pattern of a KS matches a pattern of features, the KS is said to be applicable. It will be further triggered and its output (*i.e*. a new feature) will be deposited on the corresponding layer.

### The controller

A blackboard system repeats a detection/selection/execution cycle as long as some events take place on the blackboard. The occurrence of an event may be due to the creation or the modification of an entity on a layer. This event is collected by the controller, which selects the relevant KSs. When its activation pattern can be fully satisfied, a KS is declared to be applicable. The newly applicable KSs are added in the controller agenda and receive a priority. This priority depends upon the control strategy that has been selected by the user. We have currently implemented a simple depth-first strategy: the agenda is a stack so that the first KSs to be executed are the last that have been selected. However, if needed, the data structures of the Genepi agenda allow implementing more sophisticated strategies.

### The propagation-activation network

In a naïve implementation of the controller, the activation patterns of all the KSs concerned by an event must be scanned in order to decide on the applicability of the KSs. In order to greatly improve the performance of the controller, all the activation patterns are compiled into a RETE-like [22] propagation-activation network. Indeed, the problem of selecting the KSs according to their input patterns is very similar to the problem of selecting the rules in a rule-based expert system according to their left-hand sides and the facts available on the working memory. A problem for which the RETE algorithm was initially designed.

### Efficiency issues: packing features

Implementing a blackboard such as the one described in Figure 3 will probably not be very efficient in practice, especially on large sequences, because of the low granularity of the entities involved (such as Starts and Stops). In this approach, each Start and Stop entity will be considered and treated independently by one or more KSs. As a consequence, there will be a lot of pending KSs in the agenda and the whole process will slow down considerably. In practice, it is more efficient to group these features into (homogeneous) sets of features and to make the KSs work with those sets instead of individual features. For instance, in Figure 3, the KS:HypoCDS that is responsible of computing the CDSs will take as input the set of all Starts and the set of all ORFs and will produce a set of all CDSs. This approach is called "feature packing". Of course, it is less elegant than the low granularity approach since most of the logic should be encoded within the KS instead of being explicitly declared in the blackboard. For instance, the fact that a CDS is defined as a maximal stretch of DNA between a Start and a Stop in the same frame will not be declared in the KS activation pattern but will be hidden within the internal code of KS:HypoCDS.

In practice both approaches may be mixed, e.g. by packing features in the lowest layers (i.e when producing large amount of raw features such as Starts, Stops and CDSs) and by working with individual features at the higher levels (*i.e*. when producing richer annotations). This approach will be further illustrated in the Results section.

### Implementation issues

Genepi has been implemented in Java and can be used either as a standalone application or as a library embedded into larger applications. The system can be easily extended by adding new Java classes complying with the API. Some KSs maybe associated to external executables (e.g. Blast [23]) through the use of Unix-shell calls. The current KS toolbox includes methods for finding prokaryotic ORFs, tRNAs and RBSs as well as BlastP for scanning databases. It provides the basic elements to design blackboards targeted at prokaryotic genome analysis. As it will be illustrated in the next section, configuring a new blackboard to use already existing KSs does not require any programming skill.

## Results

The prototype has not been developed with the ambition to overcome existing automatic annotation systems, but to demonstrate the appropriateness of the blackboard architecture for the development of genome annotation systems, both from the knowledge engineering and the software engineering points of view. Although the system is robust enough to be used on real-size applications, it is of primary use to bioinformatics researchers who want to experiment with blackboard architectures. To this purpose we provide, in addition to the core system, a graphical user interface allowing to load a blackboard configuration, to run it (possibly step by step) and to graphically visualize the creation of features on the chromosome during the execution.

### Instantiating a genome annotation blackboard

**Figure 5:**
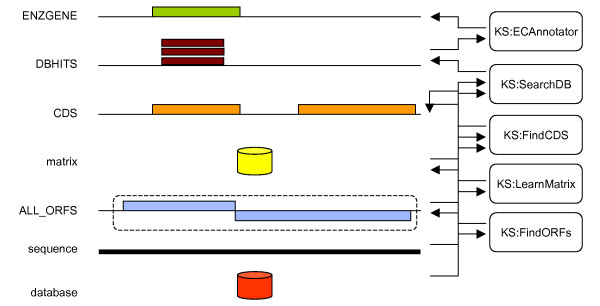
**Example of a blackboard strategy to find and annotate gene putatively encoding for enzymes**. The raw sequence is scanned to look for long ORFs (KS:FindORFs). These ORFs are then used to train a Markov model (KS: LearnMatrix) which is further used to find CDS's (KS:FindCDS). Each CDS (1_CDS) is then scanned (KS:SearchDB) against an enzyme database (database) whose entries are annotated with EC numbers. Finally, when a CDS gets sufficient matches with annotated enzymes and when the majority of these matches have the same EC number, then this EC number is transferred to the CDS annotation (by KS:ECAnnotator) to give rise to an enzymatic gene (3_ENZGENE). Note that, from the blackboard point of view, all objects that are manipulated by KSs have to be explicitly represented in layers. For instance, the database and matrix objects do have their own layers too. Also note that, for efficiency, the ORFs are not represented as individual features but are packed within a single set (dashed line).

To instantiate a blackboard, the designer must declare, in a configuration file, the number of layers, the types of the different entities, the KSs, *i.e*. their input and output patterns and their executable body. This process will be exemplified now on the real annotation strategy depicted in Figure 5. This strategy aims at finding genes on a bacterial chromosome and annotating those that putatively encode for enzymes. It works in the following way: first the chromosomal sequence is scanned (*KS:FindORFs*) for long ORFs (*ALL_ORFS*). These ORFs are used to build (*KS:LearnMatrix*) a Markov transition matrix (*matrix*). This matrix is further used to actually find (*KS:FindCDS*) *CDS*s. These *CDS*s are then checked (*KS:SearchDB*) against an enzyme database (*database*) whose entries are annotated with EC numbers (an EC number characterizes the function of an enzyme). Finally, when a CDS gets sufficient matches with annotated enzymes and when the majority of these matches have the same EC number, then this EC number is transferred to the CDS's product to eventually yield an enzymatic gene (*ENZGENE*). It is important to note that, in this example, we stopped at the gene level but one could imagine to continue using these genes in higher layers of the blackboard, representing for instance bacterial operons (sets of co-transcribed genes) or metabolic pathways (for instance, a pathway may be considered as complete when all enzymes catalyzing the biochemical reactions are present).

### Configuring the blackboard

**Figure 6:**
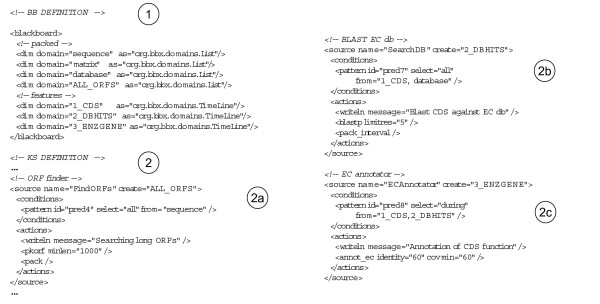
**Declaration of the blackboard implementing thestrategy depicted on Figure 5**. In order to instantiate a blackboard implementing an annotation strategy, the user just has to write an XML configuration file. This file contains two parts: one (1) for the declaration of the blackboard layers and the other (2) for the definition of the KSs. In this example, the blackboard contains seven layers (out of which three correspond to individual genomic features: CDS, DBHITS and ENZGENE). For clarity, the figure just displays an excerpt of the actual listing (the complete version can be found in the distribution of Genepi).

To configure a blackboard implementing this strategy, one has to edit an XML configuration file such as the one described in Figure 6. This file contains two parts, one for the definition of the blackboard layers (Figure 6-1) and one for the KSs (Figure 6-2). In Figure 6-1, we declare seven layers. The first four layers (i.e. *sequence, matrix, database *and *ALL_ORFS*) are implemented as lists. They represent respectively, the raw sequence, the Markov matrix, the database to scan (all of these three are singletons) and the list of all ORFs (for efficiency, these ORFs are packed within a single entity called *ALL_ORFS*). The last three layers (i.e. *CDS, DBHITS *and *ENZGENE*) are implemented as intervals (TimeLine) and represent individual features : CDSs, Blast hits associated to one CDS and validated enzyme genes respectively. The second part of the configuration file deals with the declaration of the KSs. Each KS has two parts: the activation pattern (*<conditions> *tag) and the executable body (*<actions> *tag). Moreover, the type of entities produced by a KS is specified by the *<source> *tag attribute *create*. For instance, *KS:FindORFs *in figure Figure 6-2-a, has a very simple activation pattern that reads "select any entity from layer *sequence*". It produces (a single packed) entity of type *ALL_ORFS *through the call of the executable *pkorf *declared in the *<actions> *tag. All sub-tags of the *<actions> *tag are associated to a piece of Java code. Some of them perform internal operations (e.g. *<writeln> *will write some text on the console) and others may call external executables (e.g. *<pkorf>*). When adding a new KS to the system, one will have also to define the associated tag. A more sophisticated KS example is given on Figure 6-2b. The *KS:SearchDB *is responsible of computing Blast hits associated to each CDS. Its activation pattern reads as "select all couples composed of one *CDS *and the *database*. This CDS will then be scanned (*<blastp>*) on the database and the resulting hits will be further packed (*<pack_interval>*). Finally, an example of Allen relation is given on Figure 6-2c. *KS:ECAnnotator *activates on each couple composed of one *CDS *and a set of associated hits (*DBHITS*) that is included in ("during") the *CDS*.

**Figure 7:**
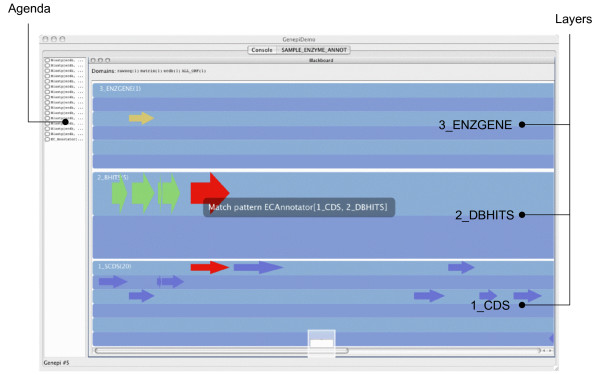
**Execution of the blackboard implemented in Figure 6**. This figure shows the Genepi graphical interface during the execution of the blackboard declared in 6. The left panel displays the contents of the agenda with several applicable KS's currently waiting for execution. The central (blue) panel displays the three layers containing genomic features. When this screenshot was taken, the system was matching the ECAnnotator activation pattern. This pattern requires one CDS and one DBHITS with a "during" relation (see Figure 6). The interface displays the activation pattern (black box in the center) and the corresponding objects (red color). After the ECAnnotator KS has been triggered, a new ENZGENE object may appear on the top layer.

As mentioned earlier, the Genepi standalone applications also provides a graphical interface allowing to follow step by step the execution of the blackboard. Figure 7 is a snapshot of this interface during the execution of the previous example. In this case, only the domain entities corresponding to individual genomic features are graphically represented (namely *CDS, DBHITS *and *ENZGENE*). As a real-sized test case, we ran this particular annotation strategy on the whole chromosome of *B. subtilis *(~4 Mb). More than 97% of the actual genes (4106) were correctly found (with 7% of over-predicted genes). 399 genes were further annotated with EC numbers, most of them (95%) beeing correct as compared to the published annotation. On the other hand, 494 genes with EC annotations remained unpredicted, indicating that this particular strategy (or its parameters) were probably too conservative. The total running time on the whole chromosome was 9 hours (on a MacBook Intel 2 GHz, 1 Gb), most of the time (99%) being actually spent in the Blast steps.

## Discussion

As already mentioned in the Background section, two main classes of architecture of automatic annotation systems can be identified. The first class, which includes pipeline, workflow and task-based systems, is characterized by a sequential method invocation scheme. The order in which the analysis methods have to be executed is static and predefined. Some variations may be accepted if alternative sequences can be described. This is often the case in task-based systems, which allow the next task to be chosen according to the results of the previous one. The main drawback of such a sequential scheme is the lack of flexibility regarding the maintenance and the modification of the system, especially when new methods are to be added. On the other hand, the end user can easily follow the execution of the system. The second class, which includes multi-agent, blackboard-based and rule-based systems, is characterized by an opportunistic method invocation scheme. The order in which the methods are called is not preset but is determined at runtime by the state of the system. The major advantage is that a new knowledge chunk (a KS, a rule or an agent) corresponding to a new method can be easily added or removed without much disturbing the other parts of the knowledge base. If the conditions of a method invocation have been properly defined, the method will be appropriately called when the corresponding state will occur in the system. Among these systems, blackboard architectures present determinant advantages: a shared working memory which is structured in layers in adequacy with the levels of hypothesis setting, a centralized control strategy which is easy to follow, an intuitive description of the methods and their activation patterns as independent knowledge sources.

### Blackboards and multi-agent systems

Multi-agent and blackboard systems are both part of the so-called distributed AI systems in which the processing capacity is distributed among multiple entities: the KSs of the blackboard systems and the agents of the multi-agent systems. The main difference lies in the way the entities communicate. In a multi-agent system, the entities, *i.e*. the agents, communicate directly one with the others by sending messages: the control is therefore also distributed. On the contrary, in blackboards, KSs never communicate directly. They find their input on the blackboard and deposit the products of their execution on the same blackboard, which can thus be seen as a shared working memory. In both cases, the advantages of the architecture result from the modularity it induces from both the software and knowledge engineering points of view. However, we consider that the existence of a shared and structured working memory, together with a central controller, produce a reasoning system which is much easier to follow and understand, and therefore easier to maintain, to tune and to extend. Moreover, the KSs are truly independent modules that can be added or removed without affecting the others. On the other hand, the multi-agent architectures are better suited to distributed environments. Despite their qualities, blackboard systems seem to be presently much less popular that multi-agent systems. An explanation for this situation has probably to be searched in the development of object-oriented techniques, which have provided the technology to efficiently implement the agents as interacting concurrent processes. In the same time, the complexity of blackboard systems increased (it was not uncommon to read about systems which included multiple blackboards and highly sophisticated control strategies) and thus lost some of their most appreciated properties.

### Advantages and drawbacks of blackboards for genome annotation

In the context of genome annotation, blackboards have several interesting features related to the way the methodological and biological knowledge can be updated. As research in bioinformatics produces new methods, they can be added to the system, wrapped as new KSs. Since KS never communicate directly but *via *the blackboard, these additions are simple because they do not interfere with the ones already integrated. From this point of view, the annotation of a genomic sequence can thus be updated after a new analysis method has been integrated. Conversely, if a sequencing error has been detected and corrected on the raw sequence, all the KS executions for which the corrected region was involved can be forgotten, their output erased from the layers on the blackboard and the inference cycle reactivated. Finally, we would like to mention another important case where the update facilities offered by a blackboard architecture could be put into play. Usually, after a first pass of fully automatic annotation, the genomic features need to be manually reviewed by experts. For instance, the Start position, the functional annotation or simply the presence of a gene may be modified. These manual modifications may have consequences on the overall annotation and need to be propagated, for instance if the modified CDS is involved in higher structures like operons or pathways. In the blackboard view, this means that the human expert plays the role of a new KS (technically the actual KS may be, for instance, a graphical editor). The propagation and update of the modification can then be handled by the architecture.

Of course, besides these advantages, blackboard (and multi-agent) systems also have some known drawbacks. The first one is the difficulty of making them do specific calculation in ordered tasks. As explained before, this is a natural consequence of their opportunistic behavior. Developers should therefore think in terms of event-driven actions rather than strictly ordered tasks. However, if such a pipeline behavior is desired then a solution is to embed the ordered tasks within a single KS. Indeed, the <action> part of a KS can be seen as a small pipeline. Of course, this leads to a less declarative system where a part of the annotation strategy becomes hidden in the KS. Depending upon the problem to be solved, there is therefore a tradeoff to find between "pure" blackboard and pipeline behavior. Another known, more technical, difficulty is related to the debugging of the system. Again, because of the event-driven method invocation scheme, it may be sometimes difficult to pinpoint the source of a potential problem.

## Conclusion

The question of the reliability of bioinformatics software takes a slightly different form depending whether one considers a single piece of software or more complicated systems such as integrated platforms. In the first case, as long as the software correctly implements algorithms that are well known and understood, the software designers may consider that the results do not require to be further explained or justified. On the other hand, for genome annotation platforms, the execution of sequence analysis algorithms merely provide clues that have to be confronted, filtered and merged according to some methodological knowledge. This knowledge can be either directly provided by the user or formally expressed and integrated into the system. However, the possibility to formally express this knowledge, as rules, objects, tasks or any other modeling entities, does not mean that the resulting system will yield pertinent results. Indeed, this highly depends upon the expertise of the designers and the results may be further discussed and possibly refuted by the end users. In this context, we believe that an annotation system should not only allow the formal expression and integration of the methodological knowledge; it must also provide facilities for the user to follow and understand the annotation process, and to tune, adapt or even refute the content of the methodological knowledge base. The blackboard architecture appears to offer most of these software and knowledge engineering properties.

## Availability and requirements

The Genepi protoptype has been implemented in Java and is freely available for download at the following url : . The distribution includes all the java sources as well as blackboard samples. The core system and graphical interface run on any platform supporting JavaVM. It has been tested on Linux and MacOSX. Some KSs (like Prokov or Blast) need external executables. These executable are provided in the distribution for MacOSX and Linux platforms.

## Authors' contributions

AV and FR initiated the project. SDD, DZ and FR designed the architecture and software requirements. SDD wrote most of the Java code and AV provided the external toolbox. All authors participated in testing the software and in editing and proofreading the manuscript. All authors read and approved the final manuscript.

## References

[B1] Stein L (2001). Genome annotation: from sequence to biology. Nature Reviews Genetics.

[B2] Rogic S, Ouellette B, Mackworth A (2002). Improving gene recognition accuracy by combining predictions. Bioinformatics.

[B3] Borodovsky M, McIninch J (1993). GeneMark: Parallel Gene Recognition for both DNA Strands. Computers & Chemistry.

[B4] Durand P, Medigue C, Morgat A, Vandenbrouck Y, Viari A, Rechenmann F (2003). Integration of data and methods for genome analysis. Curr Opin Drug Discov Devel.

[B5] Vallenet D, Labarre L, Rouy Z, Barbe V, Bocs S, Cruveiller S, Lajus A, Pascal G, Scarpelli C, Médigue C (2006). MaGe: a microbial genome annotation system supported by synteny results. Nucleic Acids Res.

[B6] Hoon S, Ratnapu KK, Chia J, Kumarasamy B, Juguang X, Clamp M, Stabenau A, Potter S, Clarke L, Stupka E (2003). Biopipe: a flexible framework for protocol-based bioinformatics analysis. Genome Research.

[B7] Curwen V, Eyras E, Andrews TD, Clarke L, Mongin E, Searle SM, Clamp M (2004). The Ensembl automatic gene annotation system. Genome Research.

[B8] Oinn T, Addis M, Ferris J, Marvin D, Senger M, Greenwood M, Carver T, Glover K, Pocock MR, Wipat A, Li P (2004). Taverna: a tool for the composition and enactment of bioinformatics workflows. Bioinformatics.

[B9] Médigue C, Rechenmann F, Danchin A, Viari A (1999). Imagene: an integrated computer environment for sequence annotation and analysis. Bioinformatics.

[B10] Gaasterland T, Sensen CW (1996). Fully automated genome analysis that reflects user needs and preferences. A detailed introduction to the Magpie system architecture. Biochimie.

[B11] Bryson K, Luck M, Joy M, Jones DT (2000). Applying Agents to Bioinformatics in GeneWeaver. Cooperative Information Agents IV, Lecture Notes in Artificial Intelligence.

[B12] Bryson K, Loux V, Bossy R, Nicolas P, Chaillou S, van de Guchte M, Penaud S, Maguin E, Hoebeke M, Bessieres P, Gibrat JF (2006). AGMIAL: implementing an annotation strategy for prokaryote genomes as a distributed system. Nucleic Acids Res.

[B13] Decker K, Khan S, Schmidt C, Situ G, Makkena R, Michaud D (2002). Biomas: A multi-agent system for genomic annotation. International Journal of Cooperative Information Systems.

[B14] Möller S, Leser U, Fleischmann W, Apweiler R (1999). EDITtoTrEMBL: a distributed approach to high-quality automated protein sequence annotation. Bioinformatics.

[B15] Carver N (1997). A Revisionist View of Blackboard Systems. Proc 1997 Midwest Artificial Intelligence and Cognitive Science Society Conference.

[B16] Erman LD, Hayes-Roth F, Lesser VR, Reddy DR (1980). The Hearsay-II Speech Understanding System: Integrating Knowledge to Resolve Uncertainty. ACM Computing Surveys.

[B17] Engelmore R, Morgan T (1988). Blackboard Systems.

[B18] Hayes-Roth B, Buchanan B, Lichtarge O, Hewett M, Altman R, Brinkley J, Cornelius C, Duncan B, Jardetzky O (1986). Protean: Deriving protein structure from constraints. Proc AAAI 1986 Fifth National Conference on Artificial Intelligence.

[B19] Terry A, Engelmore RS, Morgan AJ (1988). Using Explicit Strategic Knowledge to Control Expert Systems. BlackBoard Systems.

[B20] Suzek BE, Ermolaeva MD, Schreiber M, Salzberg SL (2001). A probabilistic method for identifying start codons in bacterial genomes. Bioinformatics.

[B21] Allen JF (1984). Towards a general theory of action and time. Artificial Intelligence.

[B22] Forgy C (1982). A Fast Algorithm for the Many Pattern/Many Object Pattern Match Problem. Artificial Intelligence.

[B23] Altschul SF, Madden TL, Schaffer AA, Zhang J, Zhang Z, Miller W, Lipman DJ (1997). Gapped BLAST and PSI-BLAST: a new generation of protein database search programs. Nucleic Acids Res.

